# Bedside rationing by general practitioners: A postal survey in the Danish public healthcare system

**DOI:** 10.1186/1472-6963-8-192

**Published:** 2008-09-22

**Authors:** Sigurd MR Lauridsen, Michael Norup, Peter Rossel

**Affiliations:** 1 Unit of Medical Philosophy and Clinical Theory, Institute of Public Health, University of Copenhagen, Øster Farimagsgade 5, 1014 Copenhagen, Denmark

## Abstract

**Background:**

It is ethically controversial whether medical doctors are morally permitted to ration the care of their patients at the bedside. To explore whether general practitioners in fact do ration in this manner we conducted a study within primary care in the Danish public healthcare system. The purpose of the study was to measure the extent to which general practitioners (GPs) would be willing to factor in cost-quality trade-offs when prescribing medicine, and to discover whether, and if so to what extent, they believe that patients should be informed about this.

**Methods:**

Postal survey of 600 randomly selected Danish GPs, of which 330 responded to the questionnaire. The Statistical Package for the Social Sciences (SPSS, version 14.0) was used to produce general descriptive statistics. Significance was calculated with the McNemar and the chi-square test. The main outcome measures of the study were twofold: an assessment of the proportion of GPs who, in a mainly hypothetical setting, would consider cost-quality trade-offs relevant to their clinical decision-making given their economic impact on the healthcare system; and a measure of the extent to which they would disclose this information to patients.

**Results:**

In the hypothetical setting 95% of GPs considered cost-quality trade-offs relevant to their clinical decision-making given the economic impact of such trade-offs on the healthcare system. In all 90% stated that this consideration had been relevant in clinical decision-making within the last month. In the hypothetical setting 55% would inform their patients that they considered a cost-quality trade-off relevant to their clinical decisions given the economic impact of such trade-offs on the healthcare system. The most common reason (68%) given for not wanting to inform patients about this matter was the belief that the information would not prove useful to patients. In the hypothetical setting cost-quality trade-offs were considered relevant significantly more often in connection with concerns about costs to the patient (86%) than they were in connection with concerns about costs to the healthcare system (55%; p < 0.001).

**Conclusion:**

Although readiness to consider cost-quality trade-offs relevant to clinical decisions is prevalent among GPs in Denmark, only half of GPs would disclose to patients that they consider this relevant to their clinical decision-making. The results of this study raise two important ethical problems. First, under Danish law physicians are required to inform patients about all equal treatments. The fact that only a few GPs would inform their patients about all of the relevant treatments therefore seems to contravene Danish law. Second, it is ethically controversial that physicians act as economic gatekeepers.

## Background

How to allocate healthcare fairly when not all medical needs can be met constitutes a major international health-political challenge. In Denmark the topic of priority-setting within healthcare entered the public agenda during the mid-1990s when the Danish Council of Ethics published a report[1] calling for a public debate on how to prioritize healthcare; but since then the subject has hardly been given any systematic public attention. The Danish Institute for Rational Pharmacotherapy, however, has made repeated efforts to create awareness about the costs of pharmaceuticals, especially among general practitioners (GPs). Recently, the Danish Board of Technology has also tried to reintroduce priority-setting to the public agenda[2]. The impact of this report on the public debate and on the allocation of healthcare remains to be seen. For the moment, however, there is no ongoing Danish debate on priority-setting.

The focus of this article is on priority-setting undertaken by Danish GPs at the clinical level. Priority-setting at the clinical level is often called *bedside rationing*. In an influential article Peter Ubel and Susan Goold have defined bedside rationing in the following manner:

Bedside rationing is the withholding by a physician of a medically beneficial service because of that service's cost to someone other than the patient. Three conditions must be met, in our view, before a physician's action qualifies as bedside rationing. The physician must 1) withhold, withdraw, or fail to recommend a service that, in the physician's best clinical judgment, is in the patient's best medical interests; 2) act primarily to promote the financial interests of someone other than the patient [including an organization, society at large, or the physician himself or herself]; and 3) have control over the use of the medically beneficial service[3].

GPs in Denmark today do not appear to be being strongly encouraged to undertake bedside rationing by healthcare regulations or official professional ethics codes, though certain drivers in this direction can certainly be detected. To begin with, in their original report on priority-setting the Danish Council of Ethics touched upon the issue of setting priorities at the clinical level and favoured granting physicians a certain amount of clinical autonomy in this respect [2]. Second, in their professional oath (Lægeløftet) physicians promise to apply their skills to the benefit of both society and their fellow human beings[4]. Third, and perhaps most importantly, GPs are required by their contract with the state to assist their region in ensuring economically responsible use of reimbursable prescriptive pharmaceuticals [5].

Although no regulation clearly requires Danish GPs to perform bedside rationing, weak encouragement can be detected. Bedside rationing is, however, controversial. It raises important questions about the way in which physicians should balance their professional duties to their patients and the healthcare system [6]. Two central considerations are in play: should physicians ration at the bedside, and if so, should they disclose this fact to their patients?

About the first consideration, it has been argued that rationing is necessary, and that since physicians are best able to recognize appropriate cases for rationing they should sometimes ration at the bedside. Efforts have been made, moreover, to find ways of involving physicians in rationing while shielding the fairness and trust that are central to the patient-physician relationship [7,8]. It has been claimed, of course, that bedside rationing should be avoided because it undermines the physician-patient relationship [9-11].

The second consideration concerns whether physicians should be implicit or explicit about rationing, i.e. whether patients should be routinely informed about rationing when it occurs. Some claim that openly acknowledged rationing should be avoided since it creates dissatisfaction among physicians and patients [12] and prompts social conflict over resource allocation [13]. Others argue that unacknowledged rationing is undesirable because it is inconsistent with the ideals of communication [8], informed consent and democratic participation [14-16].

Although there are studies of physicians' [17-21], patients' [22], and citizens' [23-25] attitudes to rationing, data on GP attitudes to bedside rationing within primary care are limited. To date there have been no studies of the question whether GPs are willing to inform their patients about bedside rationing, and no investigation of the reasons GPs would give for not informing patients (where they would not).

The aim of this article is twofold. First, we try to establish the proportion of GPs today who consider it acceptable to trade-off a treatment's health effect or side-effects against its cost given the treatment's economic impact on the healthcare system. Second, we measure the extent to which those GPs who do consider such trade-offs acceptable would inform their patients about this.

To investigate GP attitudes we presented participants with an ethical dilemma, *mainly *of hypothetical design. This dilemma was designed so as to allow us to investigate whether GPs solely attend to the interests of their patients when they prescribe medicine, or whether they also act as economic gatekeepers who steward the resources of the healthcare system.

In the remainder of this article trade-offs between effect or side-effects and cost are referred to as *cost-quality trade-offs*.

## Methods

### Study design and participants

The study proceeded in two parts: in the main study a questionnaire was mailed to a random sample of 600 GPs drawn from the Danish Medical Association's department of registration. The questionnaire, which was in Danish, was accompanied by a letter explaining the purpose of the study and that it was voluntary to participate. It also guaranteed confidentiality. Non-responders received up to three reminders. Subsequently, a one-page questionnaire was sent to all non-respondents to the first questionnaire. The first part took place in January 2007; the second in February 2007.

### Main questionnaire

The main questionnaire was constructed on the basis of theoretical considerations. It evolved following a series of four semi-structured in-depth interviews with GPs partly recruited through personal contacts and partly selected randomly from a list of all GPs in Copenhagen. The interviews, which were conducted by SL, were taped, and the tapes were carefully monitored so that common themes reported by the respondents could be located. Most of the participants in the interviews mentioned treatment of hypertension and high cholesterol as examples where they might consider trading cost off with quality of care. Against this background two hypothetical cases were constructed.

The first concerned hypertension. It was described as follows. "A 41-year-old woman goes to her GP. Examinations have repeatedly shown that her blood pressure is too high and non-pharmacological initiatives have proven ineffective. A number of medical treatments exist. There is general agreement that these are identical in effect but differ in their side-effects".

The second case concerned high serum cholesterol. "A 65-year-old man suffering from high serum cholesterol visits his physician for a check-up. An examination shows that, despite treatment with 40 mg. simvastatin, he continues to have an LDL above 5. A new treatment is planned".

After being presented with these cases, the participants were given information about the typical pharmaceuticals for hypertension and high serum cholesterol (low density lipoprotein [LDL] > 5 mmol/liter in spite of daily treatment with 40 mg simvastatin). They were also told about the costs of the various treatments, both to the patient and to the healthcare system.

**Figure 1:**
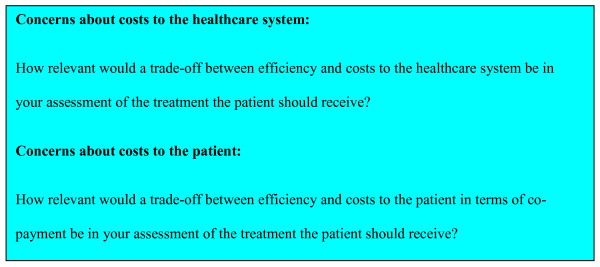
The two central questions.

All participants were asked to indicate on a four-point ordinal scale how relevant a trade-off between the treatment's health effect and side-effects and its cost would be in their clinical decision-making – given concern about the patient's costs (the patient's co-payment) and given concern about costs to the healthcare system (Figure 1).

They were also asked which of the specified alternative treatments they would spontaneously inform their patients about. Participants who indicated that they would not disclose all treatments were asked to indicate, again on a four-point ordinal scale, the importance of the following three claims in this decision: (i) The information is of no use to patients; (ii) I have no wish to enter into a discussion with my patients; and (iii) I have no time to give the information. Participants were also given the opportunity to list other reasons for not providing information.

Participants who considered a cost-quality trade-off relevant to clinical decision-making in the hypothetical cases were asked whether they would inform their patient about this. Those who would not inform their patients were asked, once more, to indicate the relevancy of the three claims listed above for this decision on a four-point ordinal scale. Those participants who claimed that a cost-quality trade-off would not be of any relevance to their clinical decision-making were asked which of the following three reasons was important in this decision: (i) I focus exclusively on the health gains of the patient; (ii) I believe that all of the specified treatments in the scheme are equally beneficial; and (iii) I based my decisions on other considerations.

Besides these questions, we asked all participants how often, on a four-point ratio scale, cost-quality trade-offs had, in general, been relevant to their clinical decision-making over the last month.

Finally, all participants were asked to specify their age, sex and number of years as GP, together with the locality of their practice.

Before the study proper was conducted, the questionnaire and the covering letter were tested in five pilot interviews with GPs conducted by SL. Some of the respondents from the first series of pilot interviews were included in a new series of interviews. The rest were randomly selected. This led to some changes in formulations and graphics. Further testing in a pilot survey of twenty randomly selected GPs did not prompt any additional changes.

### Questionnaire for non-respondents

A follow-up one-page questionnaire was sent to non-respondents to the main questionnaire (N = 267). This asked questions about how relevant, given the overall needs of the healthcare system, they consider cost-quality trade-offs to be in clinical decision-making, and questions about the provision of information about this to patients.

### Ethics

The participants were guaranteed anonymity. In Danish law, no approval of the study by a research ethics committee was required.

### Statistical analysis

The Statistical Package for the Social Sciences (SPSS, version 14.0) was used to produce general descriptive statistics. Significance was calculated with the McNemar test and the chi-square test.

## Results

### Respondents

Of the 600 questionnaires dispatched in the main study, 330 were completed and returned (55% response rate). Three questionnaires were undeliverable. Of the respondents, 211 (64%) were men and 117 (36%) women (two respondents did not specify their sex). The mean age was 53 years (four people did not specify their age). 46% of the sample worked in the eastern part of Denmark and 52% worked in the western part. 2% did not specify which part of the country they worked in.

The distribution of age, sex, and locality (i.e. eastern versus western part of Denmark) did not differ from the distribution in the general population of GPs in Denmark (data not shown).

### Bedside rationing

The majority of participants considered trade-offs between a treatment's quality and its costs relevant to clinical decision-making (Table 1). Trade-offs were considered relevant significantly more often when they were connected with concerns about costs incurred by the patient than they were in connection with concerns about costs to the healthcare system (McNemar test, p < 0.001).

**Table 1:** Percentage of GPs who consider trade-offs between costs and health effects* based on concerns about costs to the patient and costs to the healthcare system to be relevant to clinical decision-making (N = 327)

**Concern for, %**	Very relevant	Somewhat relevant	Slightly relevant	Not relevant
Patient	52	34	9	4
Healthcare system	34	41	19	6

*There was no significant difference between how relevant participants thought it to be to trade-off costs and side-effects (hypertension) and costs and effects (cholesterol) out of concern of the healthcare system nor out of concern for the patient.

Missing data: 3 participants

90% of participants (296 of 330) reported that, out of economic concern about the healthcare system, a cost-quality trade-off had been relevant to their clinical decision-making within the last month.

### Information

As can be seen in Table 2, significantly fewer respondents would inform patients about their attitude to cost-quality trade-offs when that attitude was based on concern about costs to the healthcare system than would do so when costs to the patient were at issue (McNemar test, p < 0.001). 

**Table 2:** Percentage of GPs who inform patients of their belief that, in view of concerns about costs to the patient and costs to the healthcare system, they consider trade-offs between costs and health benefits to be relevant to clinical decision-making

**Concern for, %**	Treatment	Information about trade-off	*P value
Patient (N = 312)	Effect	85	P < 0.045
Patient (N = 310)	Side-effect	80	
System (N = 305)	Effect	55	P < 0.028
System (N = 296)	Side-effect	50	

*McNemar test used to assess whether physicians have the same pattern of information when they make trade offs in cases of side-effect and cases of effect

Missing data: up to 12 participants

**Figure 2:**
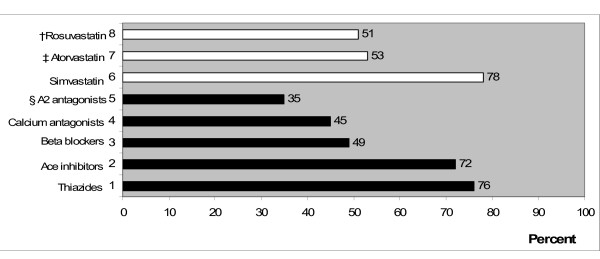
**Percentages of the extent to which physicians inform patients about various antihypertensive and cholesterol lowering treatments (N = 330). **The treatments marked with †, ‡, and § indicate the most expensive therapies.

Participants were generally less prone to disclose information about expensive treatments than they were to disclose similar information about cheaper ones (Figure 2).

### Reasons for non-disclosure

The belief that the 'information would not be useful to patients' was most frequently reported as the principal reason for not informing patients about treatments; it was also most frequently reported as the principal reason for not informing patients about cost-quality trade-offs (Table 3).

**Table 3:** Reasons GPs judged to be the most important for not informing patients about treatments and making trade-offs between cost and health benefit

	**Side-effects**		**Effects**	
**Most important reason for not informing**	No info treatment (N = 223)	No info trade-off (N = 132)	No info treatment (N = 160)	No info trade-off (N = 128)
Information not useful to patients	63	59	55	68
Various other reasons	25	19	29	15
Unwillingness to enter discussion with patients	7	11	11	8
Lack of time	5	12	6	9

Missing data: up to 51 participants

The second most reported reason for not providing information, described as 'various other reasons', included reasons the physicians themselves listed. Most of these reasons were in fact more or less blank statements that disclosure would not be useful, or brief explanations of why the physician believed that information would not be useful to patients. Thus the statements incorporated phrases such as "I do not believe the patient can use the information" and "I wish to avoid confusing the patient".

### Reasons for not making trade-offs

A small number of participants (19) said that they would not consider a cost-quality trade-off based on economic concern about the healthcare system relevant to clinical decision-making. These respondents all stated that they focused exclusively on the health outcomes for the patient, and of them 21% (4 of 19) explained that they would not make trade-offs because the specified treatments were all equally beneficial, while 26% (5 of 19) said they had other reasons for not making trade-offs.

### Determinants of reported rationing

We failed to demonstrate any association between physicians' age, sex, locality, and years in practice and the degree of reported rationing.

### Results of the non-respondent survey

In the non-respondent survey, 87% (96 of 110) of the participants believed that a cost-quality trade-off, based on economic concern about the healthcare system, would in some cases be relevant to clinical decision-making. This result did not differ significantly from the result of the main study (chi-square test). 20% (19 of 94) would not tell their patients that the relevant trade-off had influenced their clinical decision-making. Significantly more participants in the non-respondent survey than in the main study would inform patients of their belief that a cost-quality trade-off was relevant (chi-square test p < 0.001).

## Discussion

The present study reinforces the idea that GPs and those working in health policy need to address two important ethical questions. First, the widespread willingness of GPs to perform bedside rationing is ethically controversial. Such rationing has been criticized by both physicians and bioethicists [9,9-11], though, of course, some observers consider it ethically unobjectionable[8,26]. So: how ethical is bedside rationing? What are its morally questionable features?

Second, the finding that physicians are minded to inform their patients about their engagement with cost-quality trade-offs to only a limited degree may be equally controversial. Although implicit or undisclosed rationing has its defenders [12,27], recent studies show that patients and citizens prefer to be informed about whether or not aspects of clinical provision are subject to rationing by physicians [22,23]. More generally, undisclosed rationing has been characterized as morally inappropriate [15,16,28]. To this last point one may respond that, in point of fact, our study does not measure physicians' attitudes towards undisclosed rationing. The participants reporting that they would not inform their patients that they consider a cost-quality trade-off relevant to clinical decisions may choose not to do this because ultimately they would not ration. It is perfectly possible that they would consider cost-quality trade-offs relevant to clinical decision-making, but that this consideration would be trumped by other factors they deem clinically relevant. If, at the end of the day, they do not ration, why should they inform their patients that they consider a cost-quality trade-off *relevant *but not *decisive *in making their clinical decisions? To this we have two responses:

First, as stated above, only 35% of participants in the case of hypertension, and only up to 53% in the case of cholesterol, would even inform patients about the expensive treatments. This seems to indicate that GPs do not merely consider cost-quality trade-offs relevant to clinical decision-making but actually engage in such trade-offs without informing patients that this is what they are doing.

Second, if one accepts that physician and patient will ideally make clinical decisions together (shared decision-making), then it remains problematic that GPs retain information relevant to decision-making. How can patients ever be truly involved in the decision-making process if they are deprived information relevant to the decisions of the physician?

The results of this study, we believe, raise two important ethical problems. First, under Danish law physicians are required to inform patients about all equal treatments[29]. The fact that only a few GPs would disclose all relevant treatments seems to contravene this legal obligation. Second, it is ethically controversial that physicians act as economic gatekeepers.

Our finding call, therefore, we believe, for a broad discussion of undisclosed bedside rationing in healthcare involving both health professionals and the public.

### Strength and weaknesses

We now turn to indicate strengths and weaknesses of the study. We believe it to be an advantage of our approach that it is case-oriented: the obligation to consider a specific case may make physicians recognize attitudes they were not aware of beforehand. We also believe it is a strength of the study that it is based on a self-administered questionnaire rather than on interviews. Studies show that self-administered enquiries yield higher levels of reporting on sensitive matters enquiries where the questions are put by interviewers [30]. Again, the sample of the main study was relatively large; and the response rate, though modest, was still slightly above the average response rate for physicians [31].

Our study, however, also suffers from a number of limitations. Mainly, it may seem to be a limitation that our enquiries focused heavily on hypothetical cases. Hypothetical questions constitute a sub-category of attitudinal questions. In our questionnaire participants are confronted with a type of situation to which they may not have a clear-cut attitude. Research shows that when respondents do not have a clear sense about an issue they draw on different sources or aids. They may fall back on a general impression about the category to which the specific issue relates, or they may draw on more general values [32]. This, however, does not necessarily present a problem in our study. We have been interested in measuring GPs' general willingness to engage in bedside rationing and not specifically their attitudes to the presented cases. Hence, if participants have reported *general *attitudes to bedside rationing, rather than narrower attitudes to the specific cases they have been presented with, this is only an advantage.

It would constitute a problem if participants answered in what they believed was the 'politically correct' way, rather than reporting whether they actually consider cost-quality trade-offs relevant to clinical decisions. However, it is quite unclear what the *politically *correct answers to the questions in the questionnaire would be. GPs seem required to attend to both the interests of their individual patients and the economic effectiveness of the healthcare system. This may have ensured that our participants answered more truthfully than they would have done if there had been a clear politically correct answer.

Clearly, the missing data in table 3 are far from negligible. It could be that we failed to collect these data because participants became tired of answering the same set of questions about why they do not inform patients of treatments or trade-offs.

### Other studies

A number of empirical studies have investigated the GP's role in priority-setting. The study that is most similar to ours is a recent investigation of bedside rationing among general internists carried out in five European countries [21]. In this investigation 82% of participants "agreed to some extent with rationing". 56% reported that they had rationed interventions within the last six months, and 37% reported that they "sometimes did not let their patients know about the expensive alternative".

In our study 95% of the participants reported that they considered cost-quality trade-offs serving the needs of the healthcare system relevant to their clinical decision-making. 90% reported that, with the needs of the healthcare system in mind, they had actually treated a cost-quality trade-off as relevant to their clinical decision-making within the last month. Finally, up to 55% would inform patients about their readiness to consider such cost-quality trade-offs in making clinical decisions.

In both studies, then, the majority of participants considered the rationing of care relevant; and, again in both studies, a large proportion of the participants also reported that often they often do not disclose rationing information to patients.

Unfortunately, it is not possible to make a more precise comparison, because the terminology, scales and design used in the two studies differ too greatly. (A Danish version of the survey instrument used to collect the data provided in this article is available in additional file 1.)

### Further work

Finally, it would be interesting to examine the phenomenon of bedside rationing in observational studies. This would make it possible to obtain information about whether GPs *in fact *ration at the bedside in a more direct manner (though the response-effect created by the presence of an observer may, of course, introduce a potential bias in such a study).

The divergence between the main and the non-respondent study in respect of disclosure of information to patients indicates a need for further research. The belief, widespread among physicians, that information about their readiness to factor in cost-quality trade-offs when making clinical decisions is not useful to patients suggests a need for further investigation also – it would of interest, clearly, to know whether such information is in fact regarded as useful by the patients.

## Conclusion

The results of this study showed that most GPs would consider a trade-off between a treatment's cost and its quality relevant to their clinical decision-making, in view both of the costs to the healthcare system and the costs to the patient. Approximately half of the participants would disclose to patients their belief that cost-quality trade-offs are relevant to clinical decision-making given the cost of treatment to the healthcare system. More physicians would inform their patients about trade-offs where the relevant costs were to be borne by the patient than would do so where the costs were to be met by the healthcare system. The secondary, non-respondent survey supported the finding, made in the main study, that most participants consider trade-offs relevant to clinical decision-making when those trade-offs are connected with concerns about costs carried by the healthcare system.

## Abbreviations

SL: Sigurd MR Lauridsen; MN: Michael Norup; PR: Peter Rossel.

## Competing interests

Sigurd Lauridsen is an industrial PhD student (for further information concerning this PhD program see ) and as such is sponsored by the Danish Ministry of Science, Technology and Innovation and AstraZeneca A/S, Denmark.

Michael Norup and Peter Rossel have no competing interests.

## Authors' contributions

SL drafted the design and the manuscript. He participated in the analysis of the data and conducted the pilot interviews. MN has participated in the design of the study, the analysis of the data and helped draft the manuscript. PR helped conceive the study. He participated in the design of the study and helped draft the manuscript. All authors read and approved the final version.

## Pre-publication history

The pre-publication history for this paper can be accessed here:



## Supplementary Material

Additional file 1 **A Danish version of the survey instrument used to collect the data provided in this article is uploaded as a PDF file under the file name "questionnaire s.lauridsen".b** Click here for file
