# Supplementary material for: Bedside rationing by general practitioners: A postal survey in the Danish public healthcare system
Source: BMC Health Serv Res. 2008 Sep 22;8:192. doi: 10.1186/1472-6963-8-192 (PMC2567318; doi:10.1186/1472-6963-8-192)
Supplement: Additional file 1 — A Danish version of the survey instrument used to collect the data provided in this article is uploaded as a PDF file under the file name "questionnaire s.lauridsen".b [file 1472-6963-8-192-S1.pdf]

DET SUNDHEDSVIDENSKABELIGE FAKULTET
KØBENHAVNS UNIVERSITET

Spørgeskema

Lægelige overvejelser vedrørende information og prioritering

Afdeling for Medicinsk Videnskabsteori

1. Sygehistorie: En 41 årig kvinde med for højt blodtryk

En 41 årig kvinde henvender sig til egen læge. Gentagne undersøgelser viser, at hun har for højt blodtryk og ikke-farmakologiske tiltag har vist sig virkningsløse. Der findes en række medicinske behandlinger mod for højt blodtryk.

**(1) Vil du uopfordret informere patienten om følgende behandlingsmuligheder:** (besvar venligst alle spørgsmålene)	Ja	Nej
(a) A2-antagonister	☐	☐
(b) Ace-hæmmere	☐	☐
(c) β-blokkere	☐	☐
(d) Calciumantagonister	☐	☐
(e) Thiazider	☐	☐

Hvis du ikke informerer om alle behandlinger gå videre til 2, gå ellers til 4

**(2) Hvor relevante er følgende overvejelser for at undlade at informere om alle behandlingerne:** (besvar venligst alle spørgsmålene)	Slet ikke Relevant	Lidt Relevant	Noget Relevant	Meget Relevant
(a) Jeg mener ikke, at patienten vil kunne bruge informationen til noget	☐	☐	☐	☐
(b) Jeg ønsker ikke at gå ind i en diskussion med patienten om, hvilken behandling hun skal have	☐	☐	☐	☐
(c) Jeg har ikke tid	☐	☐	☐	☐

(d) Jeg har andre grunde til ikke at informere. Nævn de vigtigste:______________________________

____

(3) Anfør bogstavet ud for din absolut vigtigste overvejelse:_______

(4) I det følgende bedes du svare på, hvor relevant en afvejning mellem bivirkninger og omkostninger vil være for din vurdering af hvilken behandling patienten bør have.
I nedenstående skema er de økonomiske omkostninger forbundet med de forskellige behandlinger anført

Behandling	Pris per år for patienten	Pris per år for sundhedssystemet
A2-antagonister	1054 kr	1037 kr.
Ace-hæmmere	270 kr	0 kr.
β-blokkere	135 kr.	0 kr.
Calciumantagonister	175 kr.	0 kr.
Thiazider	302 kr.	0 kr.

**(5) Hvor relevant vil en afvejning mellem bivirkninger og omkostninger for patienten i form af egenbetaling være for din vurdering af hvilken behandling patienten bør have?**	Slet ikke Relevant	Lidt Relevant	Noget Relevant	Meget Relevant
	☐	☐	☐	☐

Hvis denne afvejning slet ikke er relevant for dit valg gå til 9, gå ellers videre til 6

**(6) Vil du uopfordret informere om, at en afvejning mellem bivirkninger og omkostninger for vedkommende i form af egenbetaling indgår i dit forslag til behandling?**	Ja	Nej
	☐	☐

Hvis nej gå videre til 7, hvis ja gå til 10

**(7) Hvor relevante er følgende overvejelser for at undlade at informere om denne afvejning:** (besvar venligst alle spørgsmålene)	Slet ikke Relevant	Lidt Relevant	Noget Relevant	Meget Relevant
(a) Jeg mener ikke, at patienten vil kunne bruge informationen til noget	☐	☐	☐	☐
(b) Jeg ønsker ikke at gå ind i en diskussion med patienten om, hvilken behandling hun skal have	☐	☐	☐	☐
(c) Jeg har ikke tid	☐	☐	☐	☐

(d) Jeg har andre grunde til ikke at informere. Nævn de vigtigste:________________________________

____

(8) Anfør bogstavet ud for din absolut vigtigste overvejelse:_______

Gå direkte til 10 efter at have svaret på 8

**(9) Hvis ingen afvejning mellem bivirkninger og omkostninger for patienten indgår i din vurdering, hvilken af følgende tre grunde skyldes det:** (besvar venligst alle spørgsmålene)	Ja	Nej
(a) Jeg fokuserer udelukkende på den helbredsmæssige gavn for patienten	☐	☐
(b) Jeg mener alle behandlingerne er lige gode	☐	☐
(c) Jeg baserer mine beslutninger på andre overvejelser	☐	☐

	Slet ikke Relevant	Lidt Relevant	Noget Relevant	Meget Relevant
**(10) Hvor relevant vil en afvejning mellem bivirkninger og omkostninger for sundhedssystemet være for din vurdering af hvilken behandling patienten bør have?**	☐	☐	☐	☐

Hvis denne afvejning slet ikke er relevant gå til 14, gå ellers videre til 11

	Ja	Nej
**(11) Vil du uopfordret informere om, at en afvejning mellem bivirkninger og omkostninger for sundhedssystemet indgår i dit forslag til behandling?**	☐	☐

Hvis nej gå videre til 12, hvis ja gå til sygehistorie 2

**(12) Hvor relevante er følgende overvejelser for at undlade at informere om denne afvejning:** (besvar venligst alle spørgsmålene)	Slet ikke Relevant	Lidt Relevant	Noget Relevant	Meget Relevant
(a) Jeg mener ikke, at patienten vil kunne bruge informationen til noget	☐	☐	☐	☐
(b) Jeg ønsker ikke at gå ind i en diskussion med patienten om, hvilken behandling hun skal have	☐	☐	☐	☐
(c) Jeg har ikke tid	☐	☐	☐	☐

(d) Jeg har andre grunde til ikke at informere. Nævn de vigtigste:______________________________

____

(13) Anfør tallet ud for din absolut vigtigste overvejelse:_______

Gå direkte til sygehistorie 2 efter at have svaret på 13

(14) Hvis ingen afvejning mellem bivirkninger og omkostninger indgår i din vurdering, hvilken af følgende tre grunde skyldes det:
(besvar venligst alle spørgsmålene)

	Ja	Nej
(a) Jeg fokuserer udelukkende på den helbredsmæssige gavn for patienten	☐	☐
(b) Jeg mener alle behandlingerne er lige gode	☐	☐
(c) Jeg baserer mine beslutninger på andre overvejelser	☐	☐

2. Sygehistorie: En 65 årig mand med for højt kolesteroltal

En 65 årig mand med for højt kolesteroltal er til kontrol hos egen læge. En undersøgelse viser, at han på trods af behandling med 40 mg. simvastatin forsat har et LDL over 5. Det planlægges at iværksætte en ny behandling.

(15) Vil du uopfordret informere patienten om følgende behandlingsmuligheder:

	Ja	Nej
(a) Atorvastatin 80 mg.	☐	☐
(b) Rosuvastatin 10 mg.	☐	☐
(c) Simvastatin 80 mg.	☐	☐

Hvis du ikke uopfordret informerer om alle behandlingerne gå videre til 16, gå ellers til 18

(16) Hvor relevante er følgende overvejelser for at undlade at informere om alle behandlingerne:
(besvar venligst alle spørgsmålene)

	Slet ikke Relevant	Lidt Relevant	Noget Relevant	Meget Relevant
(a) Jeg mener ikke, at patienten vil kunne bruge informationen til noget	☐	☐	☐	☐
(b) Jeg ønsker ikke at gå ind i en diskussion med patienten om, hvilken behandling han skal have	☐	☐	☐	☐
(c) Jeg har ikke tid	☐	☐	☐	☐

(d) Jeg har andre grunde til ikke at informere. Nævn de vigtigste:________________________________

____

(17) Anfør bogstavet ud fra din absolut vigtigste overvejelse:_______

(18) I det følgende bedes du svare på, hvor relevant en afvejning mellem omkostninger og effekt vil være for din vurdering af hvilken behandling patienten bør have. I nedenstående skema er de økonomiske omkostninger forbundet med de forskellige behandlinger anført

Behandling	Pris per år for patienten	Pris per år for sundhedssyste-met
Atorvastatin 80 mg.	1595 kr.	3680 kr.
Rosuvastatin 10 mg.	1346 kr.	2271 kr.
Simvastatin 80 mg.	515 kr.	36 kr.

	Slet ikke Relevant	Lidt Relevant	Noget Relevant	Meget Relevant
**(19) Hvor relevant vil en afvejning mellem effekt og omkostninger for patienten i form af egenbetaling være for din vurdering af hvilken behandling patienten bør have**	☐	☐	☐	☐

Hvis denne afvejning slet ikke er relevant gå til 23, gå ellers videre til 20

	Ja	Nej
**(20) Vil du uopfordret informere om, at en afvejning mellem effekt og omkostninger for vedkommende i form af egenbetaling indgår i dit forslag til behandling?**	☐	☐

Hvis nej gå videre til 21, hvis ja gå til 24

**(21) Hvor relevante er følgende overvejelser for at undlade at informere om denne afvejning:** (besvar venligst alle spørgsmålene)	Slet ikke Relevant	Lidt Relevant	Noget Relevant	Meget Relevant
(a) Jeg mener ikke, at patienten vil kunne bruge informationen til noget	☐	☐	☐	☐
(b) Jeg ønsker ikke at gå ind i en diskussion med patienten om, hvilken behandling han skal have	☐	☐	☐	☐
(c) Jeg har ikke tid	☐	☐	☐	☐

(d) Jeg har andre grunde til ikke at informere. Nævn de vigtigste:______________________

____

(22) Anfør bogstavet ud for din absolut vigtigste overvejelse:_______

Gå direkte til 24 efter at have svaret på 22

(23) Hvis ingen afvejning mellem effekt og omkostninger indgår i din vurdering, hvilken af følgende tre grunde skyldes det:
(besvar venligst alle spørgsmålene)

	Ja	Nej
(a) Jeg fokuserer udelukkende på den helbredsmæssige gavn for patienten	☐	☐
(b) Jeg mener alle behandlingerne er lige gode	☐	☐
(c) Jeg baserer mine beslutninger på andre overvejelser	☐	☐

	Slet ikke Relevant	Lidt Relevant	Noget Relevant	Meget Relevant
**(24) Hvor relevant vil en afvejning mellem effekt og omkostninger for sundhedssystemet være for din vurdering af hvilken behandling patienten bør have?**	☐	☐	☐	☐

Hvis denne afvejning slet ikke er relevant gå til 28, gå ellers videre til 25

	Ja	Nej
**(25) Vil du uopfordret informere om, at en afvejning mellem effekt og omkostninger for sundhedssystemet indgår i dit forslag til behandling?**	☐	☐

Hvis nej gå videre til 26, hvis ja gå til 29

**(26) Hvor relevante er følgende overvejelser for at undlade at informere om denne afvejning:** (besvar venligst alle spørgsmålene)	Slet ikke Relevant	Lidt Relevant	Noget Relevant	Meget Relevant
(a) Jeg mener ikke, at patienten vil kunne bruge informationen til noget	☐	☐	☐	☐
(b) Jeg ønsker ikke at gå ind i en diskussion med patienten om, hvilken behandling han skal have	☐	☐	☐	☐
(c) Jeg har ikke tid	☐	☐	☐	☐

(d) Jeg har andre grunde til ikke at informere. Nævn de vigtigste:______________________________

____

(27) Anfør bagstavet ud for din absolut vigtigste overvejelse:_______

Gå direkte til 29 efter at have svaret på 27

**(28) Hvis ingen afvejning mellem effekt og omkostninger indgår i din vurdering, hvilken af følgende tre grunde skyldes det:** (besvar venligst alle spørgsmålene)	Ja	Nej
(a) Jeg fokuserer udelukkende på den helbredsmæssige gavn for patienten	☐	☐
(b) Jeg mener alle behandlingerne er lige gode	☐	☐
(c) Jeg baserer mine beslutninger på andre overvejelser	☐	☐

Generelt

(29) Hvor ofte vil du umiddelbart mene, at følgende afvejninger er indgået i din beslutning om at ordinere behandling i løbet af den sidste måned:

(a) Jeg har afvejet en behandlings omkostninger for patienten mod dens helbredsmæssige gavn

Mere end 10 gange	5 – 10 gange	1 – 5 gange	Slet ikke
☐	☐	☐	☐

(b) Jeg har afvejet en behandlings omkostninger for sundhedsvæsnet mod dens helbredsmæssige gavn

Mere end 10 gange	5 – 10 gange	1 – 5 gange	Slet ikke
☐	☐	☐	☐

Baggrundsoplysninger

(30) Køn: Mand ☐ Kvinde ☐

(31) Alder: __________ år

(32) Hvor mange år har du været i praksis: __________ år

(33) I hvilken region er din praksis placeret: __________
